# Seawater-Activated Mineral Synergy in Sulfoaluminate Cement: Corrosion Resistance Optimization via Orthogonal Design

**DOI:** 10.3390/ma18112428

**Published:** 2025-05-22

**Authors:** Chuanlin Wang, Shupeng Zhou, Qingyou Ou, Junkai Liu, Ming Wu

**Affiliations:** Department of Civil Engineering and Smart Cities, Shantou University, Shantou 515063, China; clwang@stu.edu.cn (C.W.); 23spzhou@stu.edu.cn (S.Z.); 23qyou@stu.edu.cn (Q.O.); 24jkliu@stu.edu.cn (J.L.)

**Keywords:** sulfoaluminate cement (SAC), mineral admixtures, seawater corrosion resistance, orthogonal design

## Abstract

Mineral admixtures exhibit significant enhancement effects on the seawater corrosion resistance of sulfoaluminate cement (SAC). This study systematically investigates the influence mechanisms of fly ash (FA), silica fume (SF), and slag powder (SP) on the physicochemical properties of SAC-based materials. Experimental results demonstrate that FA effectively enhances the fluidity of fresh SAC paste while mitigating drying shrinkage. Under standard curing conditions, the compressive strength of SAC mortar decreases with increasing FA content, reaching optimal performance at a 5% replacement level. However, in seawater immersion environments, FA undergoes chemical activation induced by seawater ions, leading to a positive correlation between mortar strength and FA content, with the 10% replacement ratio demonstrating maximum efficacy. SF addition reduces workability but significantly suppresses shrinkage deformation. While exhibiting detrimental effects on flexural strength under standard curing (optimal dosage: 7.5%), a 5.0% SF content manifests superior seawater resistance in marine environments. SP incorporation minimally impacts mortar rheology but exacerbates shrinkage behavior, showing limited improvement in both standard-cured compressive strength and seawater corrosion resistance. Orthogonal experimental analysis reveals that SF exerts the most pronounced influence on SAC mortar fluidity. Both standard curing and seawater immersion conditions indicate FA as the dominant factor affecting mechanical strength parameters. The optimal composite formulation, determined through orthogonal combination testing, achieves peak compressive strength with 5% FA, 5% SF, and 5% SP synergistic incorporation.

## 1. Introduction

The world is currently facing a shortage of river sand and freshwater resources, with global production of Ordinary Portland Cement (OPC) reaching 4.1 billion tons annually, while approximately 1 billion tons of freshwater is consumed each year, further exacerbating reliance on these resources [1,2,3]. According to forecasts, global freshwater demand is expected to exceed supply by 40% by 2030, and the United Nations announced an unprecedented water crisis in March 2023. In the construction sector, particularly in coastal areas, utilizing marine sand and seawater to replace river sand and freshwater can effectively alleviate the shortage of construction resources. Concrete is an indispensable material in marine infrastructure projects, widely used in bridge piers, cap beams, and suspension bridge anchorages. Seawater contains a significant amount of corrosive ions, and engineering projects in marine environments are subjected to natural erosive forces such as wave action, abrasion, and tidal fluctuations [2,4,5]. Investigations have shown that concrete structures serving in marine environments typically experience minor to severe corrosion damage within a span of 12 to 16 years [6]. Therefore, enhancing the performance of concrete and extending its service life in marine infrastructure have become pressing issues that need to be addressed. Currently, OPC is the most widely used type of cement, but it has low early strength, which affects production cycles and construction schedules. Additionally, its poor corrosion resistance makes it difficult to apply in marine engineering. In contrast, sulfoaluminate cement (SAC) exhibits excellent properties such as high early strength, rapid setting, low alkalinity, high impermeability, and strong resistance to salt intrusion, earning it the title of “the ideal cement for marine engineering” [7,8,9]. Moreover, the calcination temperature of high-calcium SAC is 1350 °C, approximately 200 °C lower than that of OPC, reducing energy consumption. Depending on the composition, the carbon dioxide emissions of SAC are reduced by 25% to 35% compared to OPC [10].

However, as a new type of cement, SAC has some drawbacks such as the difficulty in controlling setting times and the tendency for late strength to decrease, which limit its application and development in the marine engineering field [11,12]. The incorporation of mineral admixtures into SAC not only reduces production costs and conserves energy but also improves the performance of SAC, enhancing the long-term strength and durability of concrete [11,13,14,15]. Mineral admixtures possess a good “filling effect”, “reactive effect”, and “micro-aggregate effect”. SAC is often blended with materials such as silica fume, fly ash, and slag powder to adjust setting times, inhibit strength reduction, and improve microstructure, thereby enhancing the overall performance of the cement [16,17,18]. Oleiwi et al. [19] demonstrated that fly ash accelerates SAC setting. With increasing fly ash content, the chemical shrinkage of the paste weakens, and the early strength also decreases significantly due to the dilution effect of fly ash and its relatively low reactivity, which hinders its performance in low-alkalinity environments. However, Ioannou et al. [20] studied the performance of a ternary cement system composed of sulfoaluminate, anhydrite, and fly ash. They found that the addition of fly ash (5%, 10%, 15%) promotes the formation of ettringite in the early stages of cement hydration, leading to increased early strength. The later strength of the cement increases gradually but remains higher than that of the cement system without fly ash. Many studies consider silica fume to be a material with high pozzolanic activity [21,22]. It has been found that silica fume is more reactive and its micro-aggregate filling effect improves the microstructure of the cement, thus enhancing the strength [23]. The effects of slag and fly ash on SAC are similar, inhibiting cement drying and shrinkage and reducing cement strength. Fly ash particles are mostly spherical, and after being melted at high temperatures, they can form a dense spherical glassy layer on the surface with many internal pores [24]. The so-called “ball bearing effect” of Class F fly ash reduces the amount of superplasticizer adsorbed by the cement, improving workability and the compatibility between cement and superplasticizers [25]. For fly ash particles with coarser granularity, higher carbon content, and larger porosity, which contain unburned carbon particles, they tend to absorb more water and superplasticizers, diminishing the effectiveness of the superplasticizers [26]. Silica fume has an extremely fine particle size, with most particles being spherical, smooth, and compact, giving it a “ball bearing” role that reduces internal friction between particles, which is beneficial for pumping and pouring during construction management [27]. Additionally, the different particle sizes of silica fume and cement can create a good particle gradation, resulting in a cementitious system with excellent workability and reducing segregation and bleeding during construction [28].

This study modifies SAC by singly incorporating fly ash, silica fume, and slag powder to investigate the effects of mineral admixtures on the setting time, fluidity, drying shrinkage, mechanical properties, and microstructure of SAC. Furthermore, the study employs orthogonal experiments to blend fly ash, silica fume, and slag powder in various combinations, testing the effects of the composite mineral admixtures on the fluidity and mechanical properties of SAC. The research also conducts a range analysis of fluidity, flexural strength, and compressive strength to identify the most significant factors affecting the performance of SAC. This approach aims to understand the complex interactions between different mineral admixtures and SAC, which can lead to the optimization of cement formulations for improved performance in marine construction applications.

## 2. Materials and Methods

### 2.1. Raw Materials

For the experiments, the cement used was L·SAC 42.5 low-alkalinity SAC produced by the Henan Dengdian Group Cement Co., Ltd. (Dengfeng, China). The fine aggregate was river sand, and the water used for mixing was tap water. The fly ash (FA) used was a premium grade with a fineness of 5000 mesh. The silica fume (SF) had a SiO_2_ content of 92%, and the slag powder (SP) was an S95 grade. The seawater used was prepared from ecological seawater salt produced by Jiangxi Yantong Technology Co., Ltd. (Ji’an, China). The main components of the mineral admixtures are presented in Table 1.

### 2.2. Specimen Preparation and Curing

**Figure 1:**
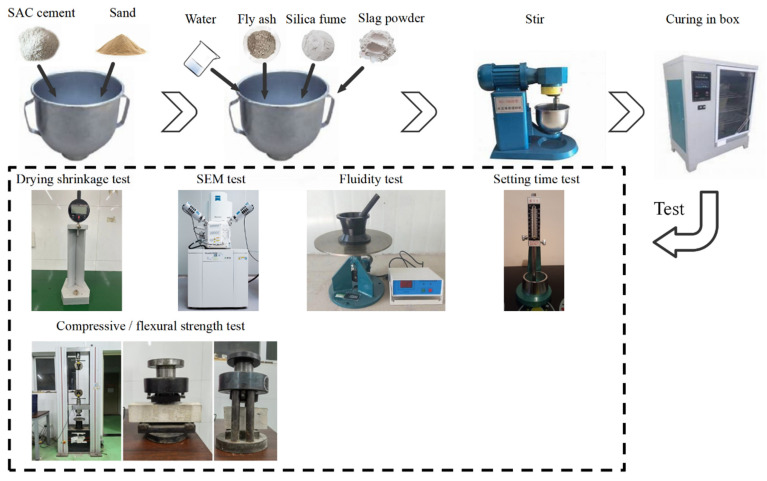
Experimental flowchart.

For the strength tests, fluidity tests, and drying shrinkage tests, the sand–cement ratio was 1.5, and the water–cement ratio was 0.45. The dosage of silica fume, fly ash, and slag powder was set at 5%, 7.5%, and 10%, respectively. According to the mix proportions, the appropriate amounts of cement, mineral admixtures, and river sand were weighed and dry-mixed uniformly. After adding the appropriate amount of mixing water, it was slowly mix at a rotation speed of 140 rpm and an orbital speed of 62 rpm for 2 min, followed by rapid mixing at a rotation speed of 285 rpm and an orbital speed of 125 rpm for an additional 2 min. The prepared cement mortar was then placed into 40 mm × 40 mm × 160 mm molds. For the SAC paste, the same water–cement ratio was used, and it was placed into 50 mm × 50 mm × 50 mm molds. After 6 h of curing, the mortar specimens were placed in a standard curing box maintained at a temperature of 20 °C ± 1 and a humidity of 95% ± 2 for 3 days. After 3 days of standard curing, half of the specimens were removed and placed into pre-prepared seawater with a tenfold concentration for immersion curing. The remaining half of the specimens continued to be cured in the standard curing box. The flexural strength and compressive strength of the specimens were tested at 3 days, 7 days, 28 days, and 56 days of curing. The specific experimental procedure is shown in Figure 1.

### 2.3. Testing Methods

#### 2.3.1. Setting Time

The setting time of the SAC paste was determined in accordance with GB/T 1346-2011 [29]. The cement paste was placed into a mold, the test needle was inserted, and the screw was tightened. The setting time was measured every 30 s until a reading of 4 mm ± 1 mm was reached, indicating the initial setting of the cement. The initial setting time is the duration from the mixing of cement with water to the initial setting. After completion, the needle was replaced with a final setting needle, and the measurement continued. When the needle penetration was 0.5 mm without leaving a trace, it indicated the final setting of the cement.

#### 2.3.2. Flowability

The flowability of the mortar was determined in accordance with GB/T 0507-2005 [30]. After cleaning the apparatus, the mold was placed in the center of the jump table, and the mortar was poured in and evenly compacted and leveled. The truncated cone mold was then lifted, and the jump table was activated for 25 cycles. The maximum spread diameter and the vertical diameter of the cement paste were measured, and their average was calculated to determine the flowability.

#### 2.3.3. Drying Shrinkage

The drying shrinkage test of the SAC mortar was conducted in accordance with GB/T 0511-2005 [31]. The prepared cement paste was placed into the mold, demolded after 6 h, and placed in a standard curing box until the corresponding age for length measurement. The average drying shrinkage rate of the three specimens was taken as the shrinkage result of the test specimen.

#### 2.3.4. Compressive/Flexural Strength

The mechanical properties of the mortar, including flexural strength and compressive strength, were determined in accordance with GB/T 17671-1999 [32]. A microcomputer-controlled electro-hydraulic servo universal testing machine with a capacity of 100 kN was used, equipped with compression and flexural testing fixtures. The loading rate for the flexural test was 0.05 kN/s, and for the compression test, it was 2.4 kN/s. The compressive strength was the average of six test blocks, and the flexural strength was the average of three test blocks.

#### 2.3.5. Scanning Electron Microscope (SEM) Images

The cured paste samples were broken, and internal fragments with a relatively flat surface, approximately 2–5 mm in size, were selected. The fragments were immersed in anhydrous ethanol to stop the hydration process and then placed in a 65 °C oven to dry for 24 h. After drying, the fragments were attached to a conductive adhesive-mounted sample stage and sent to a vacuum sputtering chamber for gold-coating treatment. The gold-coated samples were then placed in the electron microscopy equipment, a Gemini300 field emission scanning electron microscope (Oberkohen, Baden Wuerttemberg, Germany), where appropriate magnification and positions were selected to observe the microstructure of the cement.

## 3. Results

### 3.1. Setting Time

**Figure 2:**
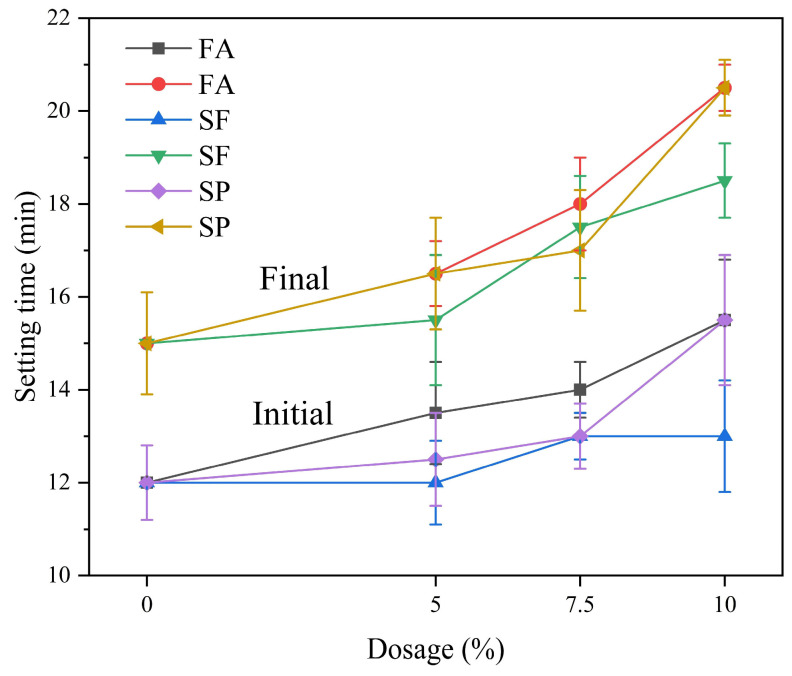
Effect of mineral admixtures on the setting time of SAC.

Figure 2 illustrates the setting time of SAC paste with different dosages of mineral admixtures at the same water–cement ratio. It can be observed that the addition of fly ash, silica fume, and slag powder all resulted in an extension of the setting time of the SAC paste. This is due to the dilution effect caused by the addition of mineral admixtures, which replace part of the cement and thus slow down the hydration reaction [33]. The composition of fly ash contains aluminosilicate glass beads, which have lower reactivity compared to cement, leading to a reduction in the hydration rate. Silica fume has a much higher specific surface area than cement, and its addition increases the water demand of the cementitious material system, thereby delaying the setting time [34]. When slag powder replaces part of the cement, it does not directly participate in the early hydration reaction of cement, which serves to delay the setting of the cementitious system and extends the setting time. Moreover, though the mineral admixtures can produce pozzolanic effect, but the pozzolanic effect is likely to occur under the condition of high temperature or longer curing time. The extended setting time can be advantageous in certain construction applications where a longer working time is required before the concrete sets. However, it is important to balance this with the need for early strength development and the overall construction schedule. The specific effects on setting time would depend on the dosage and type of mineral admixture used, as well as the environmental conditions during the curing process [35].

### 3.2. Fluidity

**Figure 3:**
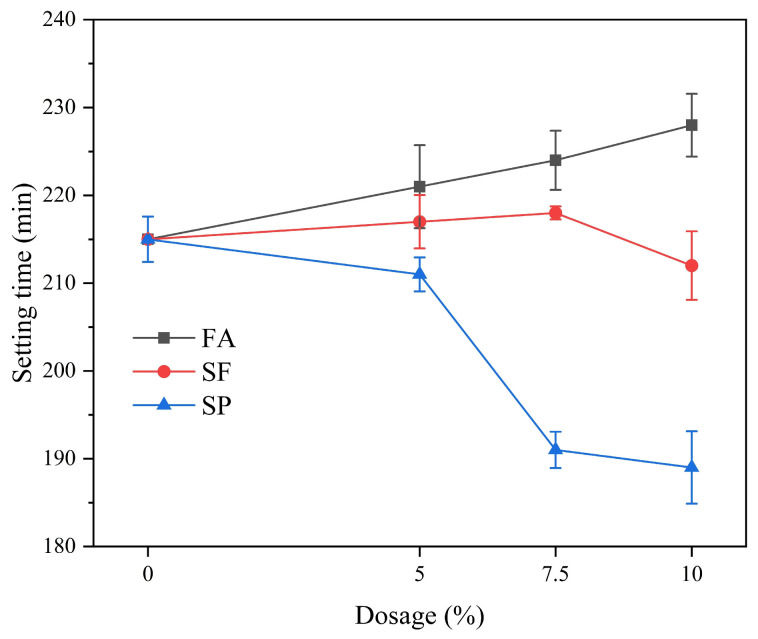
Fluidity of SAC mortar.

Figure 3 shows the fluidity of SAC mortar with different dosages of fly ash, silica fume, and slag powder. As the dosage of fly ash increases, the fluidity of the SAC mortar improves. This is because fly ash particles are typically spherical in shape, which, compared to the irregular shape of conventional cement particles, can provide better flowability. Additionally, the fine particle size of fly ash can fill the microscopic pores in the cement mortar, thus forming a more uniform paste structure and enhancing fluidity. Conversely, the fluidity of the mortar decreases significantly with the increase in the dosage of silica fume. This is mainly due to the very high specific surface area of silica fume, which means it can absorb a large amount of water, thereby increasing the mortar’s viscosity and resulting in reduced fluidity [36]. Compared to fly ash and silica fume, slag powder has a relatively minor effect on the fluidity of SAC mortar. Slag powder can effectively fill the voids in the cement, thus improving compactness. However, due to its relatively larger particle size and lower specific surface area, the filling effect on fluidity is limited.

### 3.3. Drying Shrinkage Performance

**Figure 4:**
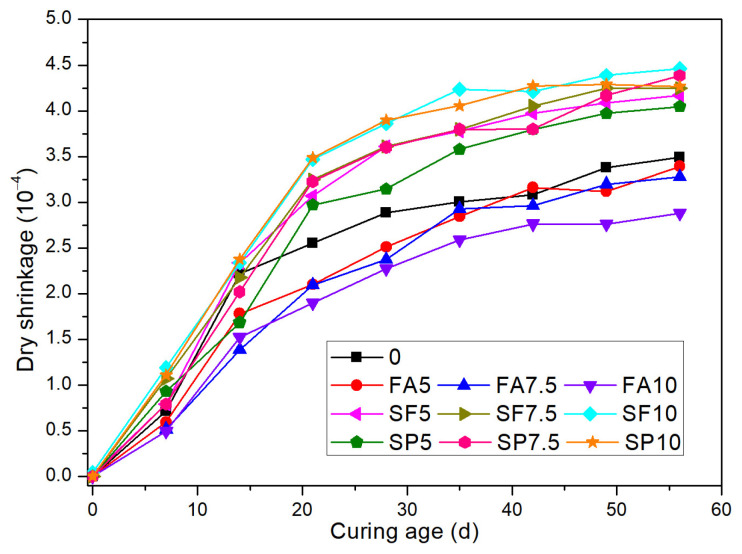
Shrinkage of SAC mortar.

Figure 4 presents the drying shrinkage changes of SAC mortar with different types of mineral admixtures at various dosages over a period of 56 days. Drying shrinkage is primarily caused by the evaporation of internal moisture from the cement-based material when its internal humidity is higher than the external environmental humidity. The figure shows that fly ash has a significant inhibitory effect on the drying shrinkage of SAC mortar, and this effect becomes more pronounced with increasing fly ash dosage. When the fly ash dosage reaches 10%, the 56-day drying shrinkage of the mortar is reduced by 17% compared to the control group without any admixture. The elastic modulus of fly ash particles is higher than that of cement particles, which can restrict the shrinkage of the cement paste within the matrix [37]. The addition of fly ash also alters the hydration process of SAC, particularly in the early stages, where its pozzolanic activity can inhibit the rapid hydration reaction of cement, thus reducing early shrinkage. Furthermore, the silica and alumina in fly ash react with calcium carbonate to form hydrated calcium silicate (C-S-H) and hydrated calcium aluminate (C-A-H), as shown in Equations (1) and (2). These compounds are beneficial for reducing micro-porosity, which in turn helps to lower the drying shrinkage of the mortar. The formation of these compounds is advantageous in reducing the drying shrinkage by enhancing the microstructure of the mortar, leading to a more dense and durable concrete. It is important to note that the optimal dosage of fly ash should be determined based on the specific requirements of the concrete application to balance shrinkage control with other performance attributes such as strength and durability.

CaCO_3_ + SiO_2_ + H_2_O → CaSiO_3_•H_2_O + CO_2_ (1)

2CaCO_3_ + Al_2_O_3_ + 10H_2_O → CaAl_2_O_4_•10H_2_O + 2CO_2_ (2)

With the increase in silica fume dosage, the drying shrinkage value of SAC mortar increases. When the silica fume dosage reaches 10%, its drying shrinkage value is 29% higher than that of the control group. Silica fume, being very fine, can fill the pores in concrete, which helps to improve the compactness of the mortar. However, silica fume significantly reduces the fluidity of the SAC paste, thus leading to a higher number of capillary pores. These capillary pores are more likely to lose moisture during the drying process, thereby resulting in greater drying shrinkage. Furthermore, the higher the content of slag powder in the mortar, the greater its drying shrinkage value. This is because the pozzolanic activity and hydration degree of slag powder are both greater than those of fly ash. Consequently, it can accelerate the rate of moisture consumption, leading to faster internal drying and thus exacerbating the autogenous drying of the cementitious material system. This increased rate of internal drying contributes to the enhanced shrinkage of the mortar [38].

### 3.4. Mechanics Performance

#### 3.4.1. The Influence of Fly Ash

**Figure 5:**
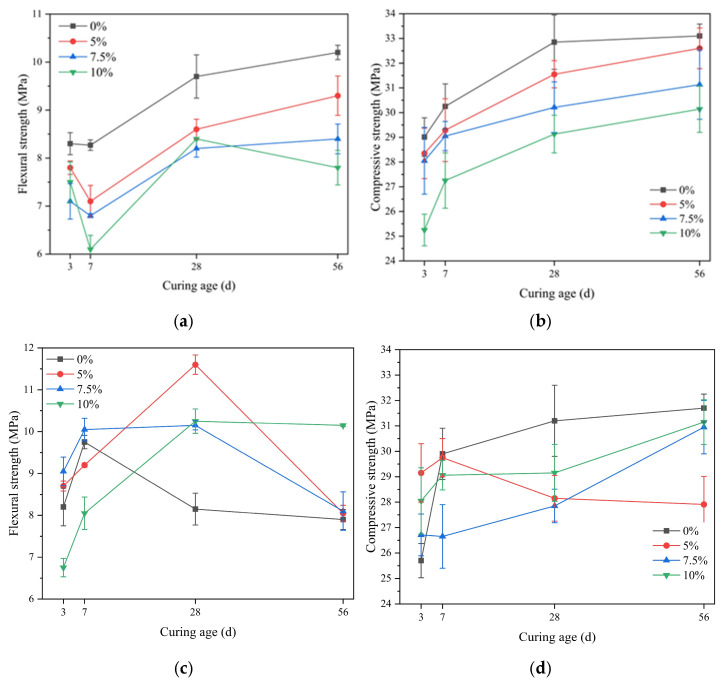
The strength of SAC with fly ash addition at various ages: (**a**) flexural strength under standard curing conditions; (**b**) compressive strength under standard curing conditions; (**c**) flexural strength under seawater curing conditions; (**d**) compressive strength under seawater curing conditions.

Figure 5 shows the strength changes of SAC mortar with different dosages of fly ash under standard curing and seawater curing conditions at various ages. The addition of fly ash significantly reduces the flexural and compressive strengths of SAC mortar under standard curing conditions, and the strength decreases as the dosage increases. SAC mortar with 5%, 7.5%, and 10% fly ash, after 56 days of curing, shows a reduction in flexural strength by 7.8%, 16.6%, and 24.6% compared to the control group, respectively. The compressive strength is reduced by 0.5%, 3.3%, and 6.3% compared to the control group, respectively, and the SAC mortar with 10% fly ash exhibits a noticeable retrogression in flexural strength. The phenomenon of reduced flexural strength in SAC mortar can primarily be attributed to the following reasons: (1) Since the activity of fly ash is lower than that of cement, its addition dilutes the cement, slows down the hydration process, reduces the content of cementitious materials, and thus leads to a decrease in mortar strength. Additionally, the SiO_2_ and Al_2_O_3_ in fly ash react with the free calcium in cement. At high dosages, this reaction consumes a significant amount of free calcium, thereby affecting the normal hydration of sulfate aluminate cement [39]. (2) Unhydrated fly ash encapsulated within the hydration products creates interfaces that reduce the continuity and density among the hydration products.

Under seawater immersion, the control group showed significant flexural strength reduction. Although the SAC mortar with fly ash also shows a retrogression in flexural strength, its later-stage strength is higher than that of the control group, and when the dosage reaches 10%, the later-stage flexural strength tends to stabilize. The compressive strength of the control group increases rapidly in the early stage and slows down in the later stage. The later-stage compressive strength of SAC mortar decreases with the reduction in fly ash dosage, and is 1.6%, 2.3%, and 11.4% lower than that of the control group, respectively. Compared to the clinker in cement, fly ash has lower activity, but under the action of alkali activators, its activity can be accelerated. Common fly ash activators include alkalis (calcium hydroxide, sodium hydroxide), sulfates (sodium sulfate), and chlorides (sodium chloride, calcium chloride), among others. The chemical components of these activators can be found in seawater and the hydration products of cement. Therefore, seawater can activate fly ash to some extent, further promoting the pozzolanic reaction of fly ash, and thus enhance the contribution of fly ash to the strength of the mortar [40].

#### 3.4.2. The Influence of Silica Fume

**Figure 6:**
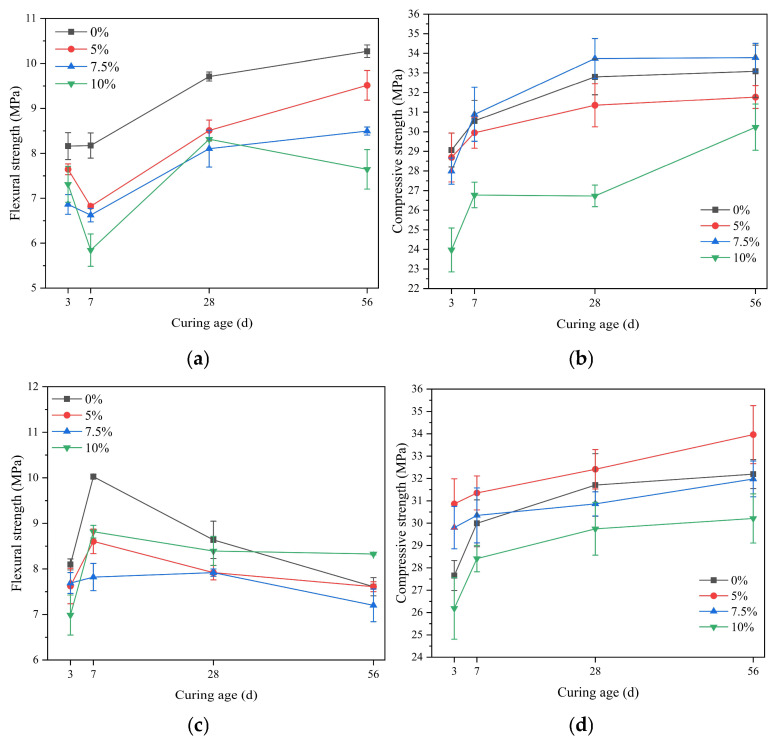
The strength of SAC with silica fume addition at various ages: (**a**) flexural strength under standard curing conditions; (**b**) compressive strength under standard curing conditions; (**c**) flexural strength under seawater curing conditions; (**d**) compressive strength under seawater curing conditions.

Figure 6 reflects the strength changes of SAC mortar with different dosages of silica fume under standard curing and seawater immersion conditions at various ages. Under standard curing conditions, the addition of silica fume at different dosages leads to a decrease in the flexural strength of SAC mortar, with the later-stage flexural strength decreasing as the dosage of silica fume increases. The 5% and 10% dosages of silica fume reduce the compressive strength of SAC mortar, while the 7.5% dosage is beneficial for the compressive strength. Under seawater immersion conditions, the early flexural strength of the mortar with silica fume is lower than that of the control group, but the 10% dosage of silica fume is more favorable for the later-stage flexural strength. With the increase in the dosage of silica fume, the compressive strength of SAC mortar gradually decreases. However, when the dosage is 5%, its compressive strength is higher than that of the control group, indicating better resistance to seawater erosion.

Silica fume has high pozzolanic activity and can participate in the hydration reaction at an early stage. Moreover, due to its large specific surface area, silica fume can effectively improve the pore structure of SAC mortar. Therefore, an appropriate dosage can effectively increase the compressive strength of the mortar and reduce the permeability of the cement paste [41]. However, an excessive dosage can lead to a relative decrease in the cement content in the system, which in turn reduces the contribution of cement to the system’s compressive strength [42]. Additionally, silica fume particles can also provide a nucleation effect for the crystallization of calcium sulfate hexahydrate (ettringite) [43].

#### 3.4.3. The Influence of Slag Powder

**Figure 7:**
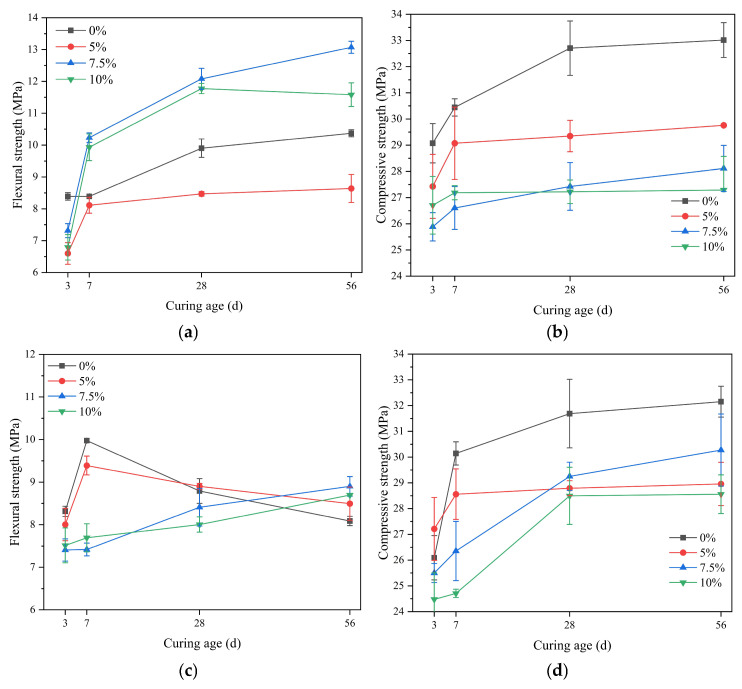
The strength of SAC with slag powder addition at various ages: (**a**) flexural strength under standard curing conditions; (**b**) compressive strength under standard curing conditions; (**c**) flexural strength under seawater curing conditions; (**d**) compressive strength under seawater curing conditions.

Figure 7 shows the strength changes of SAC mortar with different dosages of slag powder under standard curing and seawater immersion conditions at various ages. Under standard curing conditions, the addition of 7.5% and 10% slag powder is beneficial for the flexural strength of SAC mortar. However, the incorporation of slag powder is detrimental to its compressive strength, and with the increase in the dosage of slag powder, its later-stage compressive strength decreases. During the hydration process of sulfate aluminate cement, the main early hydration mineral C_4_A_3_S does not produce Ca(OH)_2_ after hydration, resulting in a very low alkalinity in the early-stage solution, which is not conducive to activating the slag powder. In the later stage, the C_2_S hydration produces less Ca(OH)_2_ compared to C_3_S, and at the same time, AFm reacts with Ca(OH)_2_ to form AFt. Since there are few components in the slag powder that can be activated, its later-stage strength is also very low. Under seawater immersion conditions, the addition of slag powder is not favorable for the compressive strength and early flexural strength of SAC mortar. However, with the increase in age, its flexural strength gradually improves and exceeds that of the control group. With the increase in dosage, the later-stage compressive strength is 11%, 6%, and 11% lower than that of the control group, respectively. The test results indicate that the addition of slag powder weakens the ability of sulfate aluminate cement to resist seawater erosion.

#### 3.4.4. The Influence of Curing Conditions

Table 2 lists the corrosion coefficient of compressive strength for SAC mortar with mineral admixtures at various ages. The corrosion coefficient of compressive strength is the ratio of the compressive strength of the test block under seawater immersion conditions to the compressive strength under standard curing conditions at the corresponding age. The addition of silica fume can increase the content of C-S-H, thereby slowing down the leaching rate of calcium and reducing the rate of deterioration. Consequently, the incorporation of silica fume is particularly beneficial for the seawater erosion resistance of the mortar, with the compressive strength corrosion coefficient exceeding 0.90 [44]. High dosages of slag powder also have a good effect on the later-stage resistance to seawater erosion. Therefore, in a marine environment, a fly ash dosage of around 10% and a silica fume dosage of around 5% are optimal, as they perform well in maintaining strength and resistance to seawater erosion.

### 3.5. SEM Analysis

**Figure 8:**
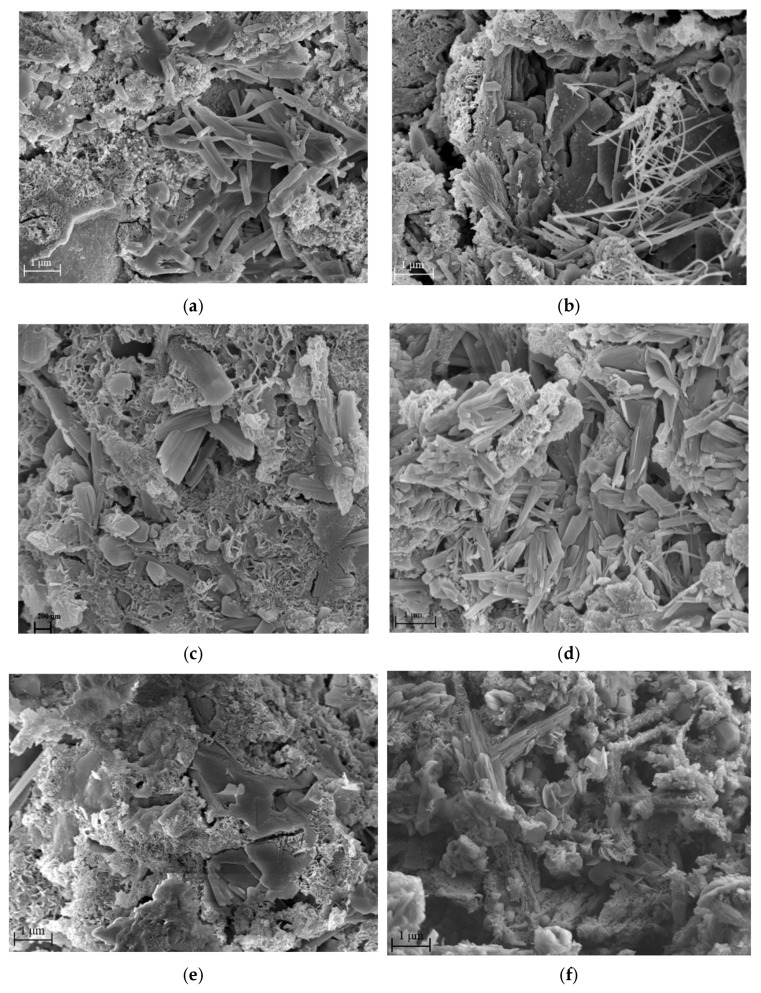
Microscopic morphology of SAC paste with mineral additives: (**a**) 5% fly ash standard curing; (**b**) 5% fly ash seawater immersion; (**c**) 7.5% silica fume standard curing; (**d**) 7.5% silica fume seawater immersion; (**e**) 10% slag powder standard curing; (**f**) 10% slag powder seawater immersion.

Figure 8 shows the microstructures of SAC mixed with 5.0% fly ash, 7.5% silica fume, and 10.0% slag powder after 56 days of curing under standard conditions and seawater immersion conditions. Comparing Figure 8a,b, under standard curing conditions, the SAC has a higher degree of hydration, producing a large number of well-developed acicular AFt and a significant amount of gel. Under seawater immersion conditions, AFt exists in a fine needle-like form, with a loose structure, and there are many strengthless flake-like AFm and unreacted spherical fly ash. This leads to a reduction in the strength of SAC with 5.0% fly ash at 56 days in a seawater environment, with a low resistance to seawater erosion, and its 56-day compressive strength corrosion coefficient is only 0.86. From Figure 8c, under standard curing conditions, the SAC paste with 7.5% silica fume has a dense structure at 56 days, with AFt being wrapped by C-S-H gel. This is due to the filling effect of the tiny silica fume particles and the conversion of calcium hydroxide into C-S-H gel through the pozzolanic effect, which fills between the cement hydration products, thereby increasing the strength of SAC. Moreover, silica fume can produce a pozzolanic effect and improve the density of the matrix. Comparing Figure 8d with Figure 8c, there are more pores in the microstructure, and the C-S-H gel does not completely wrap the calcium sulfate hexahydrate, resulting in a slightly lower compressive strength of the SAC mortar under seawater immersion conditions than under standard curing conditions. The 56-day compressive strength corrosion coefficient of SAC mortar with 7.5% silica fume is 0.94, which indicates that the SAC with silica fume still has good resistance to seawater erosion compared to the microstructure in Figure 8b. From Figure 8e,f, it can be observed that the addition of 10.0% slag powder resulted in a porous microstructure with fewer hydration products. The gel and calcium sulfate hexahydrate in the figure are less compared to fly ash and silica fume, resulting in a decrease in the compressive strength of the SAC with slag powder as the content of calcium sulfate hexahydrate, gel, and other hydration products decreases.

Long et al. [45] conducted a study and analysis of the microstructure of SAC hydration products, concluding that when the growth rate of AFt (calcium sulfate hexahydrate) is rapid, its shape is often characterized by fine needle-like crystals. Conversely, when the growth rate slows down, the shape appears as coarser, elongated columnar crystals. The amount of AFt growth increases with the acceleration of the cement hydration reaction rate, leading to a denser internal structure, reduced porosity, and thus continuous strength development. Therefore, the hydration process of SAC can be reflected by observing the amount of AFt formation and the compactness of the system. The positive effect of admixtures is mainly due to their secondary hydration, where the admixtures absorb Ca(OH)_2_ from the hydration products, reducing the system’s alkalinity and promoting the formation of acicular calcium sulfate hexahydrate. Moreover, the C_2_S in SAC clinker has high activity; under long-term curing, C_2_S continues to hydrate, producing Ca(OH)_2_, which further promotes the growth of calcium sulfate hexahydrate. The formation of AFt in the SAC system can be expressed by the following chemical equation [46]:

(3) $$C_{4}A_{3}\bar{S}+2C\bar{S}H_{2}+34H\to C_{6}AS_{3}H_{32}+2AH_{3}$$

(4) $$C_{4}A_{3}\bar{S}+8C\bar{S}H_{2}+6CH+74H\to 3C_{6}AS_{3}H_{32}$$

Variations in CaO content primarily drive microstructural changes in ettringite (AFt). At low CaO concentrations, Reaction (3) predominantly forms cylindrical or rod-like ettringite (AFt) without expansion. However, when the mass fraction of CaO is higher, AFt is formed through Reaction (4), at which point it takes on a fibrous shape and can expand. Fly ash contains free CaO, which, in the presence of evident Ca(OH)_2_, promotes the hydration of anhydrous calcium sulfate, leading to the rapid formation of AFt [47]. When the test block is immersed in seawater, the alkalinity of the system increases, resulting in the formation of mostly fine needle-like AFt, as shown in Figure 8b. In addition to the secondary hydration effect, silica fume contributes to the cement system through the nucleation of fine particles, which induces crystallization. Under certain conditions, Reaction (3) promotes the formation of AFt, and AFt appears columnar in Figure 8c, which is different from that in Figure 8a.

## 4. Influence of Composite Addition of Mineral Admixture

### 4.1. Experimental Design

This experiment employs a three-factor, three-level orthogonal test to investigate the effects of the combined addition of fly ash, silica fume, and slag powder on the fluidity of SAC mortar and its mechanics performance under seawater immersion conditions. The selected orthogonal factors and levels are shown in Table 3, and the orthogonal combinations and corresponding mix proportions are presented in Table 4. The process of making the specimens is as follows: First, the cement, river sand, and mineral admixtures are dry-mixed uniformly. Then, water is added while slowly mixing for 2 min, followed by fast mixing for another 2 min. A portion of the mixed mortar is taken out for fluidity testing, and the remaining mortar is poured into 40 mm × 40 mm × 160 mm molds and vibrated to form. The molds are demolded after 6 h. The test blocks are then placed in a standard curing box for 3 days, after which some of them are transferred to prepared seawater for immersion. The experiment tests the flexural strength and compressive strength of the specimens under three conditions: 3 days of standard curing, 56 days of standard curing, and 56 days of seawater immersion.

### 4.2. Analysis of Fluidity

**Figure 9:**
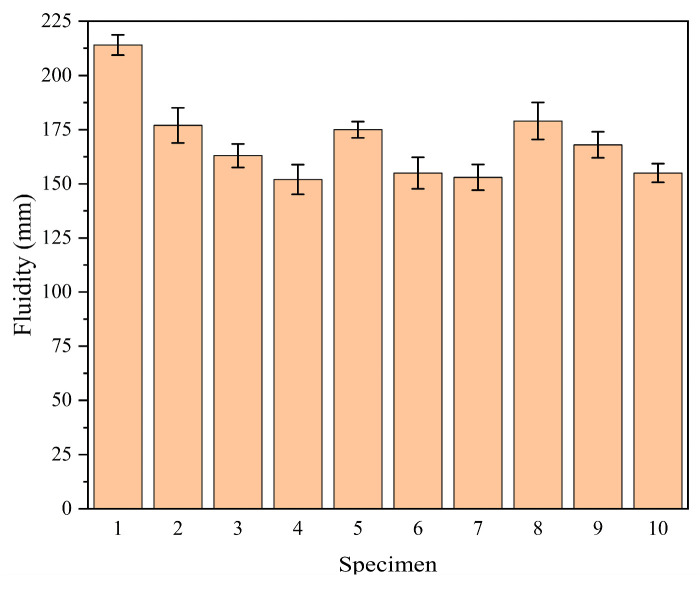
Flowability of composite mineral additives.

Figure 9 shows the fluidity values of the blank control group and the orthogonal test groups of SAC mortar. As can be seen from the figure, the combined addition of mineral admixtures reduces the fluidity of the SAC mortar. The fourth group has the smallest fluidity value, with a decrease from 214 mm in the blank control group to 152 mm, representing a 29.0% reduction in fluidity. This reduction in fluidity is likely due to the fact that mineral admixtures such as fly ash, silica fume, and slag powder can absorb part of the water used for mixing, which reduces the free water available for lubrication and thus decreases the fluidity of the mortar. Additionally, the particle size and specific surface area of these admixtures can also affect the flowability of the mixture. The smaller the particle size and the higher the specific surface area, the more likely it is that the admixture will adsorb water and affect the fluidity.

**Figure 10:**
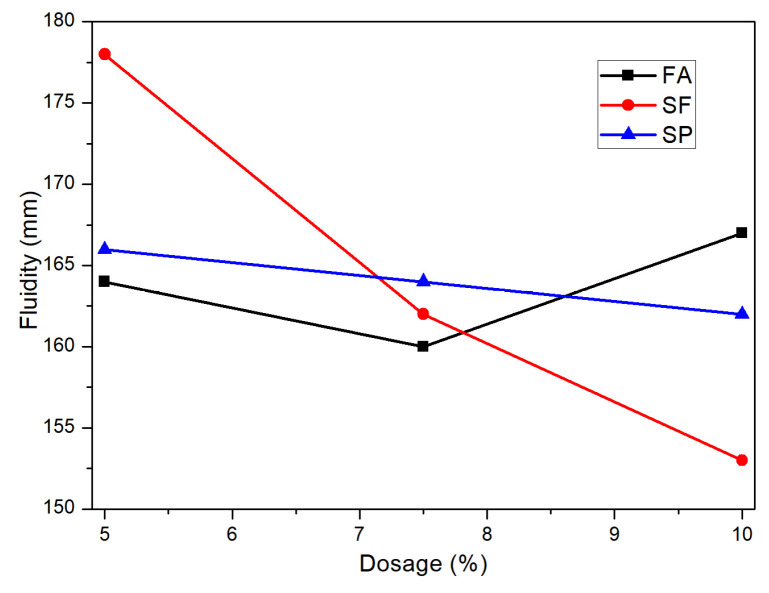
Flowability analysis effect curve diagram.

After conducting a range analysis of the fluidity values, the results are presented in Table 5. From the table, it can be observed that the order of influence of different factor levels on the fluidity of SAC mortar is as follows: silica fume dosage > fly ash dosage > slag powder dosage. The range of silica fume dosage is the largest, at 25 mm, indicating that the fluidity of the mortar decreases as the dosage of silica fume increases when combined with other admixtures. The change in dosage of slag powder and fly ash has a relatively minor impact on fluidity. Figure 10 is the effect curve of fluidity analysis for SAC mortar with different dosages of each mineral admixture. By observing the effect curve for silica fume dosage, it is evident that silica fume has the most significant impact on fluidity. When the dosage increases from 5.0% to 10.0%, the fluidity of the mortar decreases from 177 mm to 152 mm, a reduction of 14.1%. From the effect curve for fly ash dosage, it can be seen that the fluidity of the cement mortar first decreases and then increases as the dosage of fly ash increases, with an overall small change in magnitude. The trend of the effect curve for slag powder dosage is similar to that of silica fume, with the fluidity of the mortar decreasing as the dosage of slag powder increases, but the decrease is gradual, indicating that the slag powder dosage has the least impact on the fluidity of the mortar. The combination of a fly ash dosage of 10%, a silica fume dosage of 5%, and a slag powder dosage of 5% provides the highest fluidity.

### 4.3. Analysis of Flexural Strength

#### 4.3.1. Basic Data Analysis

**Figure 11:**
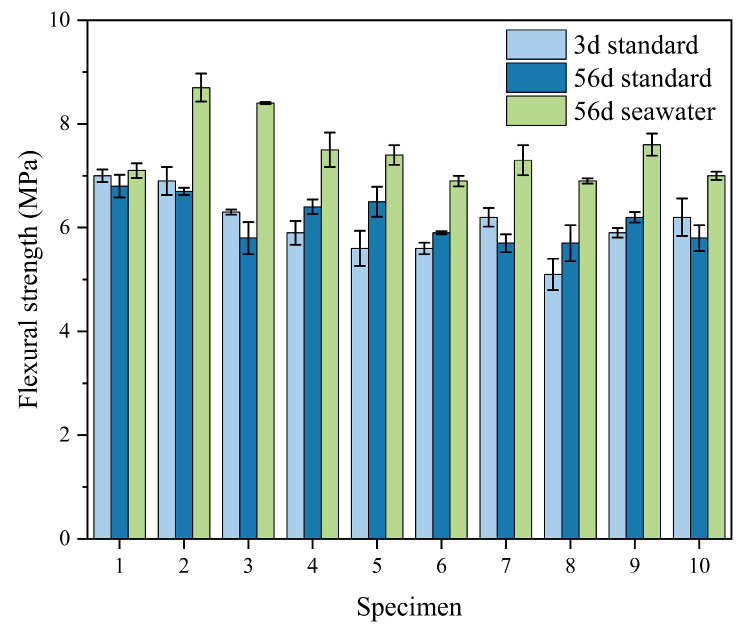
Flexural strength comparison diagram under different aging periods and environmental conditions.

Figure 11 presents the flexural strength values of the blank control group and the orthogonal test groups of SAC mortar under standard curing conditions at 3 days and 56 days, as well as after 56 days of seawater immersion. It can be observed from the figure that under standard curing conditions, the flexural strength of the mortar with combined mineral admixtures is lower than that of the blank control group, with a particularly significant reduction in early flexural strength. The eighth group’s combination of admixtures is the most detrimental to flexural strength. After the mortar specimens have been immersed in seawater for 56 days, their flexural strength is generally higher than that under standard curing conditions, and the combination of mineral admixtures is more beneficial for the flexural strength of the mortar specimens. The second group’s flexural strength is 22.5% higher than that of the blank control group. The comparison shows that the combined addition of mineral admixtures is disadvantageous to the flexural strength of cement mortar under standard curing conditions but can effectively improve its flexural strength in a seawater immersion environment.

#### 4.3.2. Range Analysis

**Figure 12:**
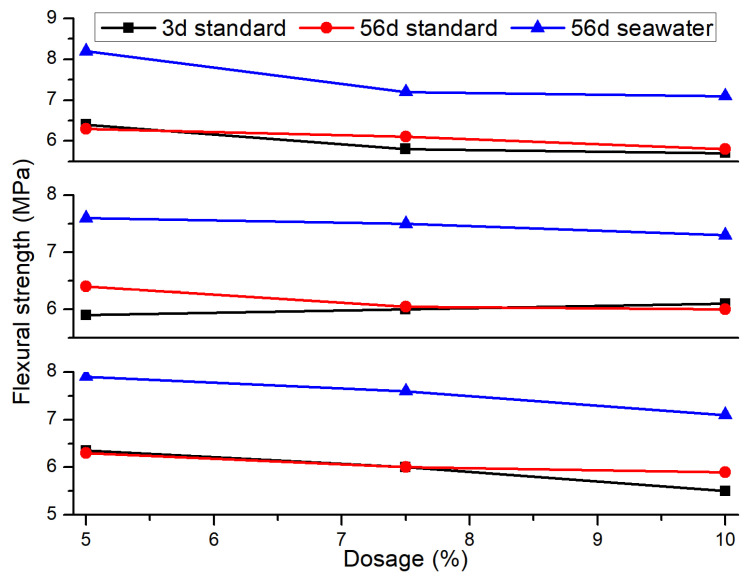
Flexural strength orthogonal effect curve.

The orthogonal effect curves of flexural strength for mortar specimens under standard curing and seawater immersion conditions are shown in Figure 12. Under both conditions, the flexural strength of the mortar decreases as the dosage of fly ash and silica fume increases. The 3-day standard cured flexural strength of the mortar increases with the increase in silica fume dosage, while the 28-day standard cured flexural strength decreases with the increase in silica fume dosage. The 28-day seawater immersion flexural strength first increases and then decreases with the increase in silica fume dosage. For mortars with mineral admixtures, their compressive strength under seawater immersion conditions is higher than that under standard curing conditions. This is because the pozzolanic reaction and micro-filling effect of mineral admixtures can easily cause shrinkage cracks in SAC, damaging its microstructure, while the seawater immersion environment improves the shrinkage of the cement mortar, thus enhancing its strength in the seawater environment. Under the 3-day standard curing condition, the order of influence of different factor levels on the flexural strength of the mortar is slag powder > fly ash > silica fume, with a fly ash dosage of 5%, a silica fume dosage of 10%, and a slag powder dosage of 5% being the optimal combination for 3-day standard cured flexural strength. Under the 56-day standard curing condition, the order of influence is fly ash > silica fume > slag powder, with a fly ash dosage of 5%, a silica fume dosage of 10%, and a slag powder dosage of 10% being the optimal combination for 56-day standard cured flexural strength. Under the 56-day seawater immersion condition, the order of influence is fly ash > slag powder > silica fume, with a fly ash dosage of 5%, a silica fume dosage of 5%, and a slag powder dosage of 5% being the optimal combination for 56-day seawater immersion flexural strength.

### 4.4. Analysis of Compressive Strength

#### 4.4.1. Basic Data Analysis

**Figure 13:**
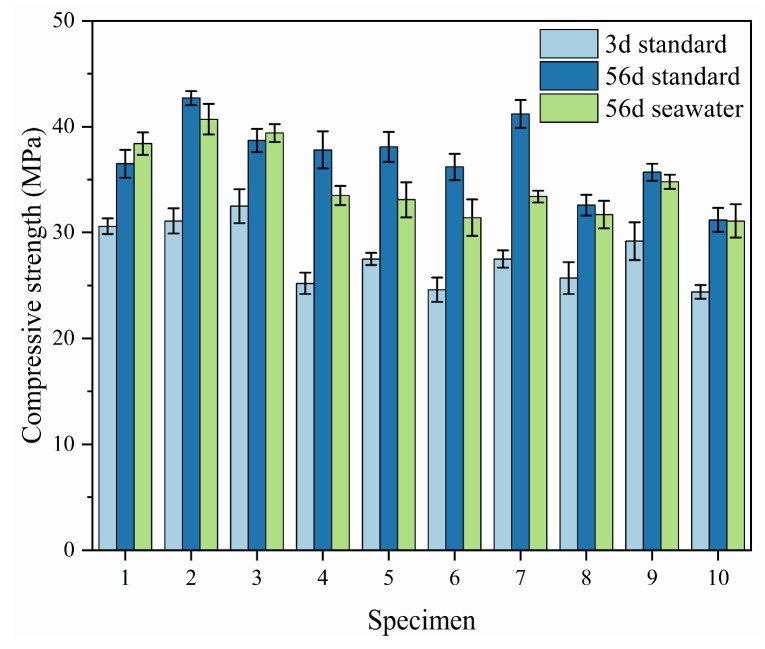
Compressive strength comparison diagram under different aging periods and environmental conditions.

Figure 13 presents the compressive strength values of the blank control group and the orthogonal test groups of SAC mortar under standard curing conditions at 3 days and 56 days, as well as after 56 days of seawater immersion. The figure shows that under standard curing conditions, the combined addition of mineral admixtures is detrimental to the early compressive strength of the cement mortar, with only the second and third groups having strength values higher than the blank control group. However, it is more beneficial for the later-stage strength, with the compressive strength of all groups except the eighth, ninth, and tenth groups being higher than that of the blank control group, showing a significant increase in later-stage compressive strength. This may be because SAC has a lower alkalinity, which is not conducive to activating the mineral admixtures. Additionally, the addition of mineral admixtures dilutes the cement content, leading to a decrease in early strength of the mortar [48]. The analysis from the figure indicates that only the second and third groups have strength values higher than the blank control group, suggesting that an appropriate dosage combination is necessary in the seawater immersion environment to increase the compressive strength of the mortar.

#### 4.4.2. Range Analysis

**Figure 14:**
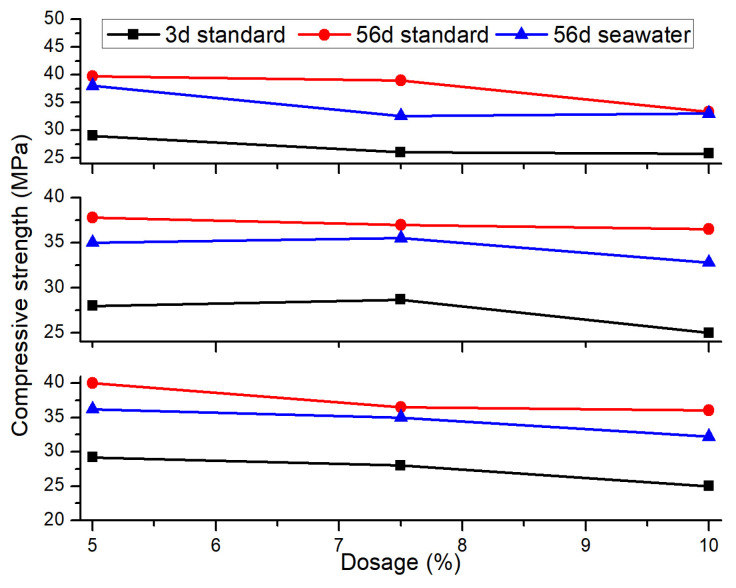
Orthogonal effect curve of compressive strength.

The orthogonal effect curves of compressive strength for mortar specimens under different curing conditions are shown in Figure 14. Under both curing conditions, the compressive strength of the mortar decreases as the dosage of mineral admixtures increases. Moreover, the strength in the seawater environment is lower than that under standard curing conditions. This is because the various salts in seawater enter into the mortar structure and affect the hydration process of cement, leading to an inhibitory effect on hydration. Since the inhibitory effect of salts on the hydration process is greater than their effect on early strength enhancement, the compressive strength shows a decreasing trend [49]. When the specimens are immersed in seawater, Mg^2+^ in the seawater is prevented from penetrating into the interior due to the densification of the cement matrix, quickly forming Mg(OH)_2_ colloids that adhere to the surface of the specimens, creating a protective layer that blocks capillary pores [50]. However, SAC contains little C_3_S and C_3_A, and the concentration of Ca(OH)_2_ in the cement is very low, with few opportunities and quantities for SO_4_^2−^ to form CaSO_4_·2H_2_O. The CaSO_4_·2H_2_O formed on the cement surface cannot penetrate the interior, thus losing the possibility of participating in the formation of AFt. Under the 3-day standard curing condition, the order of influence of different factor levels on the compressive strength of the mortar is slag powder > fly ash > silica fume, with a fly ash dosage of 5%, a silica fume dosage of 7.5%, and a slag powder dosage of 5% being the optimal combination for 3-day standard cured compressive strength. Under the 56-day standard curing condition, the order of influence is fly ash > slag powder > silica fume, with a fly ash dosage of 5%, a silica fume dosage of 5%, and a slag powder dosage of 5% being the optimal combination for 56-day standard cured compressive strength. Under the 56-day seawater immersion condition, the order of influence is fly ash > slag powder > silica fume, with a fly ash dosage of 5%, a silica fume dosage of 5%, and a slag powder dosage of 5% being the optimal combination for 56-day seawater immersion compressive strength.

## 5. Conclusions

(1) The addition of mineral admixtures exhibits a dilution effect, with the addition of fly ash, silica fume, and slag powder all prolonging the setting time of SAC.
(2) A higher dosage of fly ash is more beneficial for the fluidity of SAC mortar and has a more significant inhibitory effect on the drying shrinkage of the mortar. Under standard curing conditions, the strength of SAC mortar decreases with the increase in fly ash dosage. At low dosages, the microstructure of the paste has well-formed calcium sulfate hexahydrate (ettringite) and gel; however, in the seawater immersion environment, the fly ash is activated by the seawater components, and the strength of the mortar increases with the fly ash dosage. The microstructure at low dosages is less dense than that under standard curing. Under standard curing conditions, a fly ash dosage of 5.0% is more appropriate, while in the seawater environment, a fly ash dosage of 10.0% is more suitable.
(3) Silica fume has a high specific surface area and absorbs a large amount of water, leading to reduced fluidity of the mortar and an inhibitory effect on drying shrinkage. Under standard curing conditions, a silica fume dosage of 7.5% has an enhancing effect on compressive strength. In the seawater environment, the compressive strength of SAC mortar gradually decreases with the increase in silica fume dosage, but when the dosage is 5.0%, the compressive strength is higher than that of the blank group, showing good resistance to seawater erosion. The microstructure of the paste is similar under both curing conditions, but the microstructure in the seawater environment has more pores. Under standard curing conditions, a silica fume dosage of 7.5% is more appropriate, while in the seawater environment, a silica fume dosage of 5.0% is more suitable.
(4) Slag powder has little effect on the fluidity of the mortar and exacerbates the degree of drying shrinkage. Under standard curing conditions, slag powder is detrimental to the compressive strength of SAC mortar and, as the dosage of slag powder increases, the later-stage compressive strength decreases. In the seawater environment, the addition of slag powder is not beneficial for the compressive strength and early flexural strength of SAC mortar, and the overall effect of slag powder on resistance to seawater erosion is poor. From the microstructure observations, the addition of slag powder results in a slower hydration process of SAC under both curing conditions compared to fly ash and silica fume.
(5) When fly ash, silica fume, and slag powder are added together, under both standard curing conditions and seawater immersion, the order of influence of different factor levels on the fluidity of SAC mortar is: silica fume > fly ash > slag powder. The most significant influence on the flexural strength and compressive strength of SAC mortar is fly ash. Under standard curing conditions, the optimal combination for flexural strength is a fly ash dosage of 5.0%, a silica fume dosage of 10.0%, and a slag powder dosage of 10.0%, while the optimal combination for compressive strength is a fly ash dosage of 5.0%, a silica fume dosage of 5.0%, and a slag powder dosage of 5.0%. In seawater immersion, the optimal 56-day strength combination was 5% fly ash, 5% silica fume, and 5% slag powder.

## Figures and Tables

**Table 1:** Main chemical composition of mineral admixtures (wt%).

Ingredient	SiO_2_	Al_2_O_3_	Fe_2_O_3_	CaO	MgO	K_2_O	Na_2_O
Fly ash	46.44	38.01	3.12	7.50	0.23	0.88	0.33
Silica fume	92.18	0.23	0.09	0.99	1.83	0.31	0.05
Slag powder	34.50	17.70	1.03	34.00	6.01	-	-

**Table 2:** Compressive strength corrosion coefficient of SAC mortar at various ages.

Group	Blank	FA5	FA7.5	FA10	SF5	SF7.5	SF10	SP5	SP7.5	SP10
3 d	0.88	1.03	0.95	1.10	1.07	1.06	1.09	0.98	0.97	0.90
7 d	0.98	1.01	0.91	1.07	1.05	0.99	1.06	0.98	0.97	0.88
28 d	0.96	0.90	0.92	0.97	1.03	0.90	1.12	0.89	1.06	1.04
56 d	0.97	0.86	0.98	1.02	1.07	0.94	1.00	0.96	1.07	1.04

**Table 3:** Orthogonal test factor level table.

Level	Factor		
	A: Fly Ash Content	B: Silica Fume Content	C: Slag Powder Content
1	5.0%	5.0%	5.0%
2	7.5%	7.5%	7.5%
3	10.0%	10.0%	10.0%

**Table 4:** Orthogonal test mass proportion.

Group	Orthogonal Combination	Fly Ash	Silica Fume	Slag Powder	Cement	River Sand	Water
1	—	0	0	0	1.000	1.5	0.45
2	A1B1C1	0.050	0.050	0.050	0.850	1.5	0.45
3	A1B2C2	0.050	0.075	0.075	0.800	1.5	0.45
4	A1B3C3	0.050	0.100	0.100	0.750	1.5	0.45
5	A2B1C2	0.075	0.050	0.075	0.800	1.5	0.45
6	A2B2C3	0.075	0.075	0.100	0.750	1.5	0.45
7	A2B3C1	0.075	0.100	0.050	0.775	1.5	0.45
8	A3B1C3	0.100	0.050	0.100	0.750	1.5	0.45
9	A3B2C1	0.100	0.075	0.050	0.775	1.5	0.45
10	A3B3C2	0.100	0.100	0.075	0.725	1.5	0.45

**Table 5:** Flowability range analysis table.

Factor	Fly Ash (A)	Silica Fume (B)	Slag Powder (C)
K1	164	177	166
K2	161	162	164
K3	167	152	162
R	6	25	4
Influence degree	B > A > C		

## Data Availability

The original contributions presented in this study are included in the article. Further inquiries can be directed to the corresponding author.
